# Supplementary material for: Association between Peripheral Inflammatory Cytokines and Cognitive Function in Patients with First-Episode Schizophrenia
Source: J Pers Med. 2022 Jul 14;12(7):1137. doi: 10.3390/jpm12071137 (PMC9317024; doi:10.3390/jpm12071137)
Supplement: Supplementary file 1 [file jpm-12-01137-s001.zip › jpm-1789827-supplementary.pdf]

Table S1. Comparisons of cytokine levels according to the type of antipsychotics and sex

	TNF-α	IFN-γ	IL-1β	IL-6	IL-8	IL-10	IL-12
**Type of antipsychotics**							
Amisulpride (n=50)	8.5 (7.4-10.8)	23.4 (14.5-27.1)	2.3 (1.8-3.7)	4.1 (2.4-5.8)	4.8 (3.2-11.9)	14.6 (10.4-22.9)	4.6 (3.7-6.9)
Aripiprazole (n=30)	7.7 (5.8-9.8)	30.0 (24.3-36.2)	3.1 (2.1-3.7)	5.2 (3.4-9.6)	5.3 (3.6-21.0)	19.4 (14.1-26.6)	6.1 (4.3-8.3)
Paliperidone/risperidone (n=71)	7.9 (6.6-9.8)	23.1 (15.7-30.4)	2.4 (1.6-3.7)	3.8 (2.0-5.4)	5.4 (3.8-10.1)	13.7 (7.7-20.5)	4.4 (2.8-6.4)
Quetiapine (n=13)	9.2 (6.6-10.4)	24.6 (20.6-37.2)	2.6 (2.2-4.4)	5.2 (2.7-10.8)	6.6 (4.7-10.2)	14.7 (8.4-24.3)	5.1 (4.0-7.4)
Others (n=5)	10.0 (6.1-12.4)	26.8 (14.3-40.2)	2.9 (1.3-3.9)	2.7 (2.1-4.8)	8.7 (5.4-11.9)	14.8 (10.5-25.9)	5.6 (3.1-6.7)
None (n=5)	10.4 (7.1-13.6)	17.4 (9.9-47.0)	1.7 (0.9-4.4)	3.2 (1.5-5.9)	4.7 (3.8-6.3)	10.8 (4.5-23.2)	3.9 (2.5-7.2)
p-value	0.240	0.057	0.290	0.116	0.736	0.144	0.130
**Sex**							
Female (n=96)	8.4 (6.5-10.7)	25.9 (21.1-34.5)	3.0 (2.0-3.8)	4.3 (2.5-6.3)	5.2 (3.7-9.1)	16.7 (9.9-24.9)	5.4 (4.1-7.4)
Male (n=78)	8.4 (6.9-10.2)	22.0 (13.2-27.7)	2.2 (1.7-3.4)	3.6 (2.1-5.7)	5.4 (3.8-12.7)	13.6 (9.3-20.4)	4.3 (2.9-6.0)
p-value	0.888	0.001	0.014	0.255	0.594	0.072	0.001

TNF-α, tumor necrosis factor-alpha; IFN-γ, interferon-gamma; IL, interleukin
